# On the Utility of Evacuations and Very Low Regimen in Removing or Developing Obscure Diseases with Anomalous Symptoms

**Published:** 1817-11

**Authors:** James Johnson


### *On the Utility of Evacuations and very low Regimen in removing or developing obscure Diseases with anomalous Symptoms.* By James Johnson, M. D.

LET two physicians set out in the morning, and visit twenty of the first patients on the list of a general practitioner; and let them, after leaving each patient, arrange his complaint in a System of Nosology, or place it on a separate list to be classed after a further developement, I believe that at least ten out of the twenty would stand over for future arrangement; and that a month's observation of the other ten would displace many of them from the niche in which they first stood. In this case then, it follows, that in one half of the diseases we meet with, we are forced to work in the dark, or prescribe for the most prominent *symptoms*, till the nature of the malady be more clearly ascertained. Thus, after all our nosological systems and classifications, we find ourselves, in a majority of instances, *at the bed-side of sickness*, acting without any *fixed principle* to guide us. It is not my object in this paper to supply this principle; but to throw out some hints, and relate some cases, that may help us to avoid error in combating the symptoms of anomalous and obscure diseases.

I cannot well conceive how any important, or even unimportant organ of the body can be diseased in structure, or disturbed in function, without producing a greater or less degree of what is termed *debility*, if we except convulsions, mania, and the delirious stages of some fevers. In these, there certainly does sometimes appear preternatural strength, at least in many of the voluntary and involuntary muscles, contemporaneously with lesion of function or structure in an internal organ. But, in almost every other instance, *debility* is invariably complained of, whatever be the seat or nature of the disease. How often then does the routine practitioner prescribe for a *symptom*, when the means commonly employed for its removal must tend to aggravate the disorder? If it can be shewn then, that *debility* in obscure diseases, so far from indicating the propriety of nourishing food and tonics, is most easily removed by evacuations and low regimen— that it is, in most cases, a fallacious index, and almost always a dangerous guide for the administration of remedies, some good will be done, and some useful purpose gained.

Case 1. Mr. Whitehead, by trade a tailor, and about

27 years of age, consulted me on the evening of the 17th of December, 1815, respecting the following curious symptom, which annoyed him excessively, and rendered his life very uncomfortable. He stated, that as soon as he fell asleep, at night, he began to dream of some nauseous and disgusting object, which he fancied himself to be eating, and that the disagreeable impression caused him to start suddenly from his sleep in great agitation of mind, and accompanied by a most intolerable taste in his mouth. He was forced, each time that he thus awoke, to get out of bed, and wash his mouth repeatedly with cold water, before the nauseous taste was removed. This scene recurred several times every night. He had applied both to regulars and quacks; swallowed abundance of drugs and nostrums; but without experiencing any alteration or amendment.

He complained of *great debility,* for which he had been recommended wine and nourishing food, and in which he indulged, but without their imparting any vigour to his constitution. He was extremely irritable; startled at the least noise; was always brooding over his malady; and swallowed every nostrum that was blazoned forth in the newspapers by the Charlatan tribe. Palpitation of the heart was a very troublesome and distressing symptom. I questioned him respecting masturbation; but he firmly denied any addiction to that vice.

On placing him in a recumbent position, the left side of the thorax sounded obscurely; but the cardiac pulsations against the ribs were strong and tumultuous, and could be felt distinctly over a surface at least four inches in diameter. The pulse at the wrist was not very regular, and occasionally intermitted. The urinary secretion was scanty, and deposited a lateritious sediment. The bowels were torpid, and the intestinal secretions depraved. Abdominal pressure gave much uneasiness.

Twenty ounces of blood were taken from the arm, which he bore well. A brisk cathartic was exhibited; and his plan of living entirely altered to a low and aqueous regimen.

The succeeding night was passed in a profound sleep, without a single dream, or sensation of nauseous taste in the mouth. He could scarcely credit the circumstance when he awoke; so long had he been tormented with this singular and disagreeble symptom. I warned him, however, against this delusive security, and assured him that nothing but the most rigid regimen, and steady perseverance in the medicines I was about to prescribe, would at all preserve him in any thing like comfortable health.

He was ordered pills composed of ex. col. comp. sub. hydrargyri, and tartarized antimony, so as to keep the bowels very open; while digitalis, squill, and soda, were prescribed, to act on the urinary organs.

Under this treatment, with low diet, and only water for drink, he continued free from the symptoms above mentioned. His urinary secretion increased; the fæcal evacuations improved, and became regular; the palpitation became much less distressing; and, in short, he was in every respect a different person.

I examined the thorax a month afterwards, and thought the left side sounded better; but the cardiac pulsations were the same. Abdominal pressure gave him now much less uneasiness than formerly. He adhered with the most scrupulous exactness to all my prescriptions, both in regard to diet and medicines. He embarked for the United States of America in February 1816, carrying with him the formulæ of the medicines he had taken; and assuring me that nothing would ever induce him to change his vegetable food and aqueous drink for a more nourishing regimen.

I pretend not to define the precise nature of this man's complaint. I have stated the case with fidelity, and leave the reader to draw his own conclusions. I would venture to surmise, however, that the function, if not the structure of the *heart*, was in fault; and attentive observation has convinced me, that lesions of this organ are more productive of strange and anomalous symptoms than those of any other viscus in the human frame.

Case 2. Mr. Blake, ætat. 45, by trade also a tailor, consulted me on the 15th of April, 1816. He reports that he fell down in a fit about four or five months ago; since which, his health has been very bad. His complaints at present are—pains in the region of the heart, and throughout the abdomen. He is constipated, sallow, emaciated. No appearance of hepatic disease on examination of the right side. When placed in the recumbent posture, a distinct pulsation was visible *in epigastrio*, and could be easily and unequivocally traced some way below the umbilicus. The left side of the thorax sounds badly; no cardiac pulsation can be felt while he lies on his back; but when turned on the left side, an obscure and irregular beat is felt against the lowest margin of the ribs. He complains much of tenderness there on pressure; abdominal pressure causes very great uneasiness and anxiety, "taking away his breath," as he terms it. Says he feels a dull pain occasionally, just above the pubis. Urinary secretion rather scanty: *ægritudines somni* very distressing; pulse small and feeble; countenance extremely dejected, and says he is so weak that he can scarcely walk across the street. I recommended him to adopt a very low regimen, to drink nothing but water, and to take nearly the same medicines as in the former case.

*April* 23. He has continued the medicines, and adhered to the low regimen. He says the pain in the cardiac region is much less. He has been much purged by the medicine, and the digitalis made him frequently sick: complains of the debility as being very great. He does not start from sleep so much as formerly. On examining him to-day, no cardiac pulsation can be felt in any part of the thorax, or in any position of the body. The abdominal pulsation can still be traced below the umbilicus, but is not so visible *in epigastrio*. Abdominal pressure does not produce such a sense of suffocation. The urine is still lateritious and scanty.

*April* 25. Can sleep better, and thinks his appetite is returning. But he is very languid and dispirited. Feels a burning sensation in the region of the heart. Bowels much relaxed by the pills; urine somewhat increased. To continue the medicines; but with little hope of success.

*May* 1. He returned to-day in good spirits, stating, that he could now sleep much better; that his appetite was very keen, and longed to indulge in good food; that he could lie on either side; that his urine was in large quantity, and clear; and that he had much less pain in the region of the heart. On placing him in the recumbent posture, pressure was made on the epigastrium, but caused little or no uneasiness. The abdominal pulsation was gone, and the thorax sounded much better. *He was in a state of ptyalism from the calomel in the pills.* To continue the same medicines and diet.

*May* 4. He is still better. His appetite has been so good that he broke through the rules one day, but found he could not sleep that night. He is now very cautious. He feels much stronger. His mouth very sore.

*May* 14. Continues to improve; sleeps well on either side without starting. I could feel no abdominal pulsation on examining to-day. He has no pain in the region of the heart.

*June* 12. Having left off his medicines, and begun to indulge in animal food, he has had a partial relapse of some of the symptoms, particularly the *ægritudines somni*, depression of spirits, and sense of debility. His urine also decreased in quantity, and began again to deposit a lateritious sediment. No cardiac or abdominal pulsation can be discovered; and little of the original decubitus difficilis is complained of. He has been forced to have recourse to his former medicines and regimen, from which he always derives the greatest benefit. I saw him six months after this, and he appeared in tolerably good health; and was working at his trade.

I know not whether it may be accidental, but I have met with more cardiac affections among tailors, than among people of any one other trade. Can the bent position of the hip and knee joints, with the curvature of the body forwards, offer any obstruction to the free circulation of the blood, and predispose to affections of the heart? Is it not on this principle, that Hackney-coachmen are supposed to be more liable to popliteal aneurism than others? What was the precise nature of this man's complaint? I am sure it is more than I know. I did not suspect any hepatic affection; and yet there was little amendment till *ptyalism* took place. The full influence of mercury on the system, by opening, as it were, every pore, every secretion, every excretion, has a powerful effect, not only in reducing the quantity of the circulating fluids, but in *equalizing* the circulation itself; and thus relieving the central organ from oppression, when its function or structure is deranged.

In what manner are we to account for the urinary secretion being scanty, in many cardiac lesions; and for that dull, heavy pain and weight above the pubis, which so very frequently accompanies them? The despondency and sense of debility are accompaniments that might be naturally expected, when so important an organ as the heart is impeded in function or deranged in structure.

Case 3. A young man, on board a ship in this harbour, in the prime of life and apparently of health, complained to the surgeon that he snored so loud in his sleep as to annoy greatly all those who slept near him; and wished to know if he could have any thing to prevent this disagreeable habit. He was laughed at and dismissed. A few days afterwards, the surgeon was summoned to him in great haste, and found him in an apoplectic fit; of which he died in six hours, notwithstanding that every depletory mean was used to unload the vessels of the brain. I assisted in examining this man *post mortem.*

The integuments of the head were full of blood, and the pericranium adhered with unusual firmness to the subjacent bone. The dura mater was so inseparably adherent to the inner surface of the skull, that no force which we could use, was sufficient to raise the skull cap, without tearing the membrane off the brain. This we were obliged to do, after dividing the falx, where it is attached to the crista galli. The superior and lateral sinuses were gorged with black fluid blood. The venous system of the pia mater was in a similar state, while the arterial system appeared as if injected with red wax to the point of extravasation. The pia mater itself was every where so agglutinated, by means of its vessels, and layers of coagulated lymph, to the substance of the brain, that it could not be separated in any part. Very little water was found in the ventricles, but about an ounce was effused at the base of the skull round the medulla oblongata. The meninges covering the bases of the cerebrum and cerebellum were still more inflamed than round the hemispheres.

Now I think, that no man will deny that cerebral inflammation must have been going on, at least some days, before the fatal event took place; and it is reasonable to infer, that this anomalous symptom of extraordinary noise in his sleep was indicative of an internal affection that was little suspected, but which might probably have been removed or developed, had evacuations, *vascular and intestinal,* been promptly employed at that time.

Case 4. The very next day after the above dissection, I was called upon, in a medico-legal capacity, to investigate the cause of death in a young man who had died very suddenly and unexpectedly of a *febrile* complaint, so apparently trifling in its nature, and devoid of danger, that, till within forty-eight hours of dissolution, the surgeon had not thought it necessary to have him released from a prison, in which he was confined. *Debility* and a weak pulse prevented any active measures being employed, and completely masked the fatal approach of death. I opened the head in presence of Dr. Lara, Mr. Wilkinson, and some other medical gentlemen.

" Dura mater firmly adherent to the internal surface of the skull, and in some places lacerated by the separation. Superior longitudinal sinus filled with fluid blood, black as ink, some of which was extravasated between the dura mater and skull. The arterial and venous systems of the pia mater were completely injected with red and black blood. The hemispheres of the brain were so glued together, and to the falciform process, as to require separation by the knife. The medullary substance of the brain was every where speckled with large points of blood, which, in several places, was extravasated out of the vessels. There was a moderate effusion of water into the ventricles. The tunics of the optic nerves most beautifully injected with blood. The lateral sinuses filled with black fluid blood. The vascular system of the cerebellum was still more finely injected than that of the cerebrum. Considerable effusion of water about the base of the cerebellum and round the origin of the medulla spinalis. Extravasation of black blood under the pia mater covering the pons Varolii."

From the foregoing appearances, the medical gentlemen present were unanimously agreed, that the young man died of *phrenitis*. This case surely proves, that *debility*, in many cases, is a symptom that most emphatically calls for the lancet and other evacuations, instead of indicating the use of tonics, or of paralyzing our arms with the mephitic vapours of *typhus*.

Case 5. On the 26th of September, 1816, I was invited by Mr. Porter to inspect the body of a convict, who, while sweeping the deck of a ship, apparently in perfect health, having made no complaint to any one, was seized with a giddiness, fell down, and instantly expired. The vessels of the brain were not only turgid with blood, but *inflammation*, unequivocally marked, pervaded almost every part of the brain itself. Effusion of water had commenced, and large patches of coagulable lymph here and there glued the membranes together. I dissected the brain carefully myself, and can vouch for the high degree of inflammation.

This case surely demonstrates the *insidious* nature even of *cerebral* inflammation, and ought to put us on our guard against the evil in the slightest affections of the sensorial organ. During the Brunonian mania in fevers, how many must have fallen sacrifices to the dread of the lancet, and to the rage for diffusible stimuli! How many children are, *even now*, cut off daily by inflammation and congestion in the brain, when treated for bowel complaints by absorbents, aromatics, and opium!

Case 6. Mary Whiteheart, ætat. 20, married. June 19th, 1816.

Her friends report, that she was yesterday seized with a *fit*, but whether of an epileptic or hysterical nature cannot be ascertained by their vague description: since the fit, however, she has entirely lost the power of deglutition, the sense of hearing, and the faculty of speech. She is perfectly sensible, makes frequent efforts to speak, obeys every sign that is made to her, and points to her throat with much anxiety of countenance. She appears evidently hysterical. Her pulse is natural, except being somewhat accelerated; tongue moist; skin cool; bowels constipated. The throat, about the thyroid cartilage and gland, is unusually full and prominent; and she seems to feel pain or tenderness when these parts are handled. She is extremely docile, holding out her arm to be bled, and even insisting on holding the bason. Twelve ounces of blood were abstracted. She attempted to swallow an opening draught, but could not. A blister was applied across the throat. Nothing was perceptibly the matter with the fauces on inspection.—Eight, P. M. The blister has acted, and about half an hour ago she swallowed, though with much difficulty, a cup of tea. She cannot articulate, or hear. No buff on the blood; the countenance is more tranquil; but the pulse has risen in frequency. She has now swallowed a brisk purgative draught.

*June* 20. Blister rose well; swallowed some drink in the night. At ten o'clock this morning, the faculty of speech and sense of hearing returned in an imperfect degree. Bowels still constipated: another strong purgative exhibited.

*June* 21. She ate a hearty meal last evening, and appeared much better, but spent a bad night; and at nine this morning was seized as bad as ever. On my visiting her at eleven, she had recovered a little, and could articulate faintly; but the power of deglutition was entirely gone. The pulse is 90, the tongue foul. She makes signs to, and also complains of, the region of the liver: pressure there gives much uneasiness. A blister to the nape of the neck; and some liq. ant. tart. to be swallowed, if possible, in tea-spoonsful till vomiting is produced.

*June* 22. The blister rose, and sufficient tartrite of antimony was got down, to produce full vomiting of greenish matters; after which the power of deglutition returned in a considerable degree. To-day she is deaf; the pulse 80; tongue clean; bowels constipated. To take a scruple of jalap every four hours, till the bowels are freely evacuated.

*June* 23. No stool from four powders. To-day, the region of the liver will not bear the slightest pressure, and a constant pain there is complained of. The deglutition, hearing, and speech, considerably improved. To take pills composed of colocynth, calomel, and tartrite of antimony, every three hours, till the bowels are well opened.

*June* 24. Has taken six pills, without any effect. The eyes are quite yellow; the region of the liver very painful; but deglutition, speech, and hearing, nearly restored. A violent hæmorrhage from the nose took place last night. The vascular system tranquil to-day. A blister to the side, and the pills to be continued every four hours.

*June* 26. During the last two days, she has taken sixteen purgative pills before the bowels were freely opened. The fæces were at first extremely black and fœtid; afterwards they became yellow and copious. She is now apparently well. The hepatalgia is gone, and the organs of sense in full vigour. She is in a state of *ptyalism* from the calomel in the pills. No more medicine was exhibited.

There can be little doubt that the hepatic congestion, obstruction, irritation, or whatever name may be given to it, was the origin of the curious anomalous symptoms which first shewed themselves, and which seemed to entitle the disease to a place in the class "*Neurosis*," and to a proper quantum of *antispasmodics*. Yet bleeding, blistering, vomiting, and purging, *developed* a very unsuspected source, and, in conjunction with the influence of mercury, removed both cause and effect at the same time.

J. JOHNSON.

*St. George's Square, Portsea, Oct.* 1, 1817.

---

